# Community Structure Detection for Overlapping Modules through Mathematical Programming in Protein Interaction Networks

**DOI:** 10.1371/journal.pone.0112821

**Published:** 2014-11-20

**Authors:** Laura Bennett, Aristotelis Kittas, Songsong Liu, Lazaros G. Papageorgiou, Sophia Tsoka

**Affiliations:** 1 Centre for Process Systems Engineering, Department of Chemical Engineering, UCL (University College London), Torrington Place, WC1E 7JE, London, United Kingdom; 2 Department of Informatics, King's College London, Strand, WC2R 2LS, London, United Kingdom; Wayne State University, United States of America

## Abstract

Community structure detection has proven to be important in revealing the underlying properties of complex networks. The standard problem, where a partition of *disjoint* communities is sought, has been continually adapted to offer more realistic models of interactions in these systems. Here, a two-step procedure is outlined for exploring the concept of *overlapping* communities. First, a hard partition is detected by employing existing methodologies. We then propose a novel mixed integer non linear programming (MINLP) model, known as OverMod, which transforms disjoint communities to overlapping. The procedure is evaluated through its application to protein-protein interaction (PPI) networks of the rat, *E. coli*, yeast and human organisms. *Connector* nodes of hard partitions exhibit topological and functional properties indicative of their suitability as candidates for multiple module membership. OverMod identifies two types of connector nodes, *inter* and *intra-connector*, each with their own particular characteristics pertaining to their topological and functional role in the organisation of the network. Inter-connector proteins are shown to be highly conserved proteins participating in pathways that control essential cellular processes, such as proliferation, differentiation and apoptosis and their differences with intra-connectors is highlighted. Many of these proteins are shown to possess multiple roles of distinct nature through their participation in different network modules, setting them apart from proteins that are simply ‘hubs’, i.e. proteins with many interaction partners but with a more specific biochemical role.

## Introduction

Community structure detection is widely accepted as a means of elucidating the underlying properties of complex networks. In the standard community structure detection problem, the aim is to partition a network into disjoint communities, also known as modules, which are generally regarded as semi-independent units. In protein interaction networks, disjoint community structure detection methods have served to propose functionally coherent modules [1], [2]. However, in reality proteins may carry out more than one task or belong to more than one protein complex [3], corresponding to membership of more than one module. If disjoint communities are assumed to correspond to functional units, then overlapping communities offer a means of expressing the coordination of these functions within the context of the entire system. Consequently, relaxing the constraint of strictly non-overlapping communities in models of community structure may represent a more true to life abstraction, thus leading to a more accurate representation of cellular interactions.

The overlapping community structure detection problem is less well-defined than the standard problem and can be formulated in various ways depending on analytical requirements or user interpretation. As a result, existing approaches vary to a large degree. Methods exist based on clique percolation (CFinder [4]), local expansion and optimisation (OSLOM [5], OCG [6] and ClusterONE [7]), agent-based and dynamical algorithms (GANXiSw [8]), Approximate Minimum Degree Ordering (MOFinder [9]), normalised cut calculation (Graclus [10]), regularized sparse random graph model (RSRGM [11]), modularity optimisation (OMIM [12]), consensus clustering [13], hub duplication [14] and Markov clustering (R-MCL [15]). The first challenge is therefore to decide how to interpret the problem and subsequently to define a suitable solution procedure in relation to biological systems. Overlapping communities in protein interaction networks have been studied to derive the functional cohesion of overlapping communities compared with disjoint communities, according to enrichment of GO terms and correspondence with protein complexes [6], [7], [9], [11], [12], [15], [16]. In [6], proteins belonging to many modules in the human PPI network were found to have on average a higher node degree and node betweenness, to contain more protein domains and to be annotated with more GO terms than proteins belonging to only one module. Similarly, in [9] proteins participating in many modules in the human PPI network were found to be enriched for druggable targets according to the Druggable Genome [17]. The method proposed in [18] has been applied to a structural brain network, where each node corresponds to a brain region [19] and nodes assigned to more than one module were found to have a higher degree and higher nodal efficiency. Analogous ideas have been found in a social network modelling the spread of disease, where nodes bridging communities were found to be potential immunisation targets [20].

These studies introduce the idea that nodes with multiple module membership may play an important role within a system, either in topological or functional terms. However, despite such previous investigations, there is still no concrete definition or understanding of the nature of nodes with multiple module membership, leaving much scope for (i) the development of a solution procedure that clearly demarcates overlapping modules and (ii) the systematic investigation of links between node clustering properties and their functional potential. We address these issues by first extending previous work on network module detection based on modularity optimisation to allow for multiple module membership. We then use the clustering features of protein nodes as means to explore their topological and functional significance on system properties.

Modularity is a measure that expresses how well-defined the community structure of a network is by comparing the fraction of edges that lie within modules minus the expected value in a null model, i.e. a network with same degree distribution but edges placed at random [21]. The metric provides a natural, intuitive description of community structure for a wide range of biological applications [1], [2], [22]–[24]. Optimisation of modularity is one of the most popular methods for community structure detection (e.g. [25]–[28]) and has been applied in various solution procedures, including simulated annealing [26], [29], greedy algorithms [27], [30] and spectral methods [28]. Mathematical programming has also been used to solve modularity maximisation problems, achieving globally optimal solutions in small to medium networks [31], [32] and competitive results for larger networks [33]–[37]. Here, we extend our previous mathematical programming approaches to modularity optimisation ([31], [34], [36]) to detect overlapping modules as outlined below.

Given a *hard partition* of a network, i.e. a partition of non-overlapping communities, if each module relates to a semi-independent functionally cohesive unit, then nodes that form edges across the borders of the communities can be thought of as bridges between different functional groups. We define these nodes as *connector* nodes and we distinguish them from *isolated* nodes, that only possess links with nodes of the same community. Consequently, our proposed method takes existing modularity optimisation methodologies one step further by considering the dynamics at the borders of communities. We pose the following questions: what effect do connector nodes have on the modularity of their neighbouring modules, what parameters determine whether a connector node has a multiple module membership, what effect do these connector nodes have on the cohesion of the network and do they exhibit some biological relevance?

**Figure 1:**
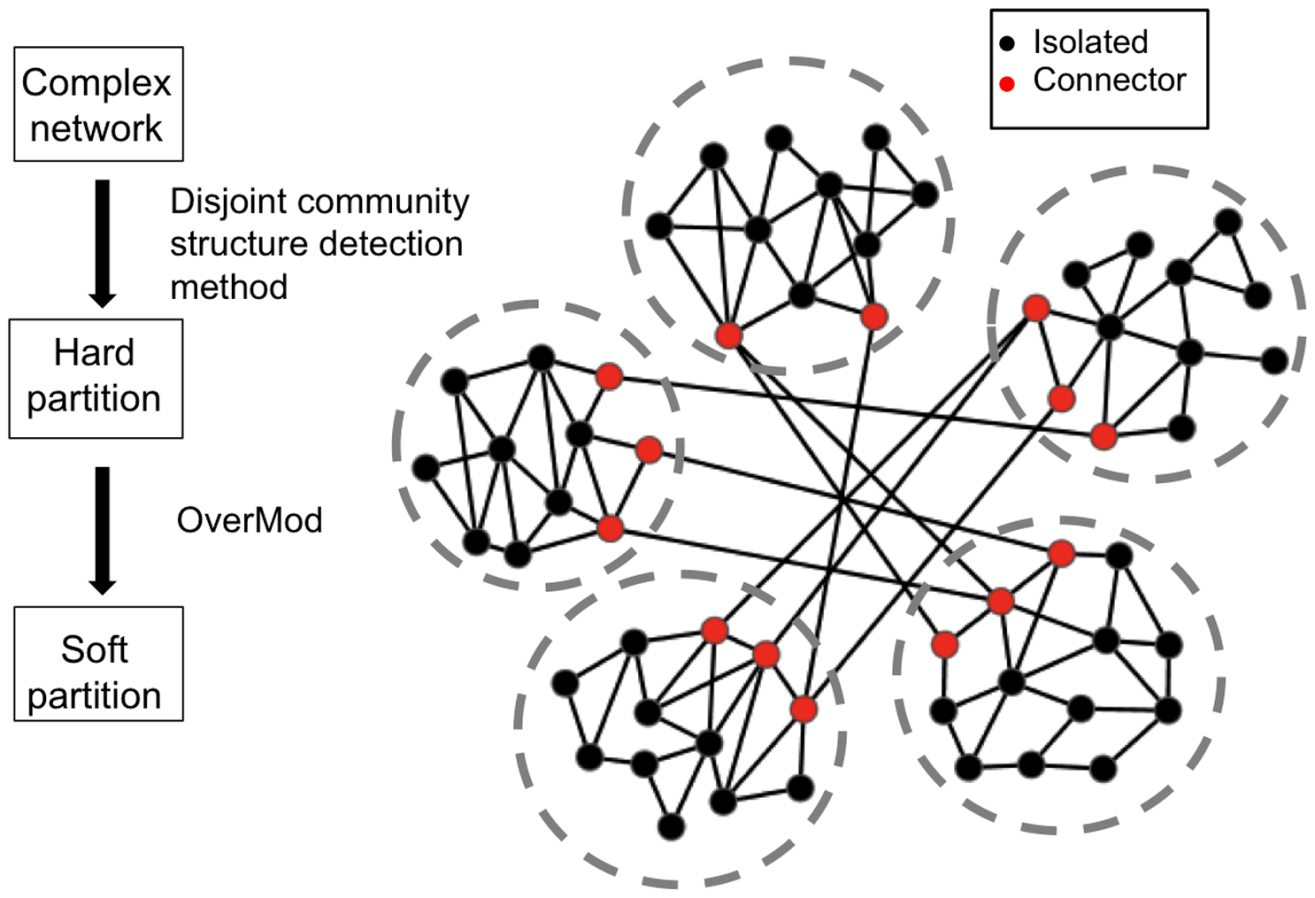
Outline of the two-stage procedure for detecting overlapping community structure. Black nodes (isolated nodes) have their module membership fixed, whereas red nodes (connector nodes) are free to be assigned to one or more modules when solving OverMod.

Our proposed two-stage procedure is outlined in Figure 1. First, a hard partition is detected by defining the disjoint communities of a network using existing well-established and tested methodologies for modularity optimisation. Through this step, connector nodes are identified and distinguished from isolated nodes. In stage two, the association of connector nodes with their neighbouring modules is considered by allowing them to be allocated to any of the modules that they interact with. Finally, connector nodes will be assigned to a module if modularity is increased and therefore they become either *inter-connectors* if they are assigned to multiple communities, or *intra-connectors* if they remain a member of a single community, resulting in a *soft partition* of the network. The transformation from disjoint to overlapping communities is achieved by a mixed integer non linear programming (MINLP) model, known as OverMod.

Our method is evaluated through the investigation of the disjoint and overlapping community structures of the rat, *E. coli*, yeast and human PPI networks. Properties of connector against isolated nodes are first examined and OverMod then determines which of the connector nodes remain assigned only to their original module (intra-connectors), and which are distributed across many modules (inter-connectors). Analysis of each category of nodes reveals their own particular characteristics pertaining to their topological and functional role in the organisation of the network. Finally, a comparative analysis of related methodologies from the literature is presented, where method performance is discussed in relation to synthetic networks and the PPI networks.

## Materials and Methods

### A Mathematical Programming Model for Transforming Disjoint to Overlapping Communities

In our previous work, modularity optimisation has been formulated as a mixed integer quadratic programming (MIQP) model [31] and a mixed integer non linear programming (MINLP) model [34], [36] to detect disjoint communities. In this work, modularity is again used as the objective function in the optimisation problem, but here node-module allocations for nodes that have no connections outside their community, known as isolated nodes, are fixed, leaving only connector nodes free to be assigned to one or more modules. The new model, OverMod, transforms a disjoint partition of a network into a partition with overlapping communities. The input required for OverMod is an undirected, weighted or unweighted network together with a hard partition of the network. The output is a set of overlapping communities. The indices, sets, parameters and variables associated with OverMod are defined below.
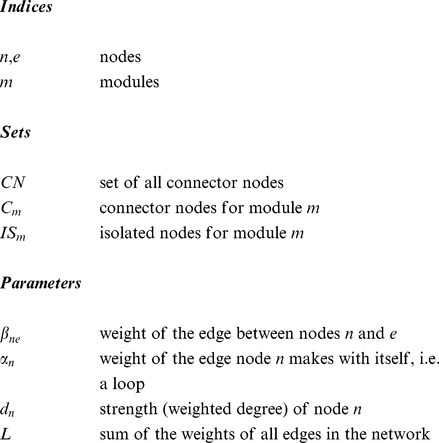





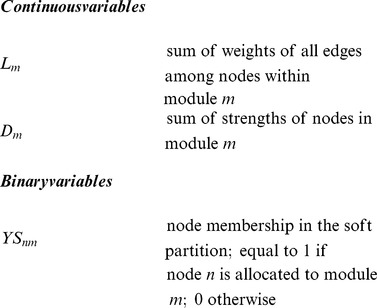



If 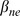 is non-zero, then an edge exists between nodes 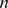 and 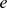 and 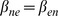. The sets 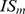 and 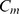 are defined according to each module, 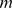, in the hard partition. 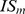 is the set of isolated nodes in module 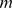; nodes which belong to module 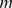 and do not interact with nodes outside of module 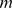. 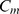 is the set of connector nodes associated with module 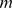; nodes in module 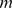 that interact with nodes in neighbouring modules or, nodes outside module 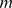 that are connected to nodes within module 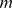.

We adopt modularity as our objective function, defined as follows:
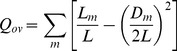
(1)where 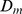 is the sum of the strengths (weighted degrees) of all nodes in module 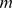, 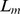 is the sum of the weights of the edges with both associated nodes belonging to module 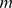 and 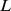 is the sum of the weights of all edges in the network. We label modularity 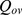 instead of simply 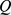 in order to distinguish it from modularity values where each node is assigned to only one module. The idea behind the approach is that since isolated nodes do not connect with nodes in other modules, they would make little or no contribution to the modularity of modules other than their own. Consequently, their module membership remains fixed and only connectors have the possibility of belonging to multiple modules in the course of the conversion procedure. In other words, for all 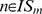, 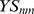 is fixed to 1, and for all 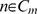, 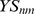 is assigned a random initial value of 0 or 1. The number of variables in the optimisation problem is therefore reduced and in turn, so is computational cost.

Equation 1 is optimised subject to the following constraints. First, 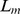 and 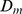 are defined as:
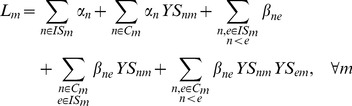
(2)and
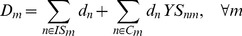
(3)where 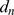 is the strength of node 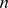 and is defined as 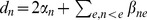. Note that OverMod accommodates self-interactions, also known as loops, 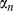.

In order to account for the overlapping aspect of the model, the following constrains each connector to belong to at least one module:
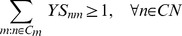
(4)


The resulting MINLP model comprises a non-linear objective function with a combination of integer and continuous variables, summarised as:

**Maximise:**

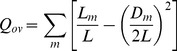
(5)


**Subject to:**

Constraints (2-4)
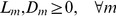
(6)




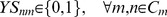
(7)


### Implementation

OverMod was implemented in GAMS (General Algebraic Modelling System) [38], where the MINLP is solved using the SBB (standard branch and bound method) mixed integer optimisation solver and CONOPT as the NLP solver. Due to the non-convex nature of the model, globally optimal solutions cannot be guaranteed. Thus, the MINLP is solved iteratively 100 times, each time with a different random initial solution, giving a approximate representation of solution space. The largest value of 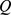 corresponds to the best soft partition. The GAMS binaries of the MINLP algorithm are available on request.

### Networks

The proposed procedure is evaluated through its application to protein-protein interaction (PPI) networks of the rat, *E. coli*, yeast and human organisms. The rat PPI network was downloaded from BioGRID (version 3.1.86, July 2012) [39]. Only interactions where both nodes were proteins of rat were retained. We consider only the main component of the network, which has 487 nodes and 572 interactions. The protein interaction network of *E. coli* was downloaded from the IntAct database (July 2013) [40], comprising exclusively interactions with a relation of type ‘direct interaction’ or ‘physical association’ that have been experimentally verified. The main connected component has 668 nodes and 846 interactions. We also consider the yeast PPI network of Collins et al. [41], from the BioGRID database. The main component of the yeast network comprises 1002 nodes and 8313 interactions. Finally, we include the main component of the human PPI network, as used in [6] and made available by the authors, which comprises 6160 nodes and 24014 interactions.

### Statistical Analysis

Various comparisons are made where the average degree, betweenness, eigenvector centrality, number of GO terms and number of protein domains of groups of nodes were calculated. Betweenness and eigenvector centrality were found using the igraph library [42] in the statistical computing environment R [43]. GO annotations for rat, *E. coli*, yeast and human were downloaded from [44], [45], [46] and [47], respectively. Each protein was mapped to all possible GO terms. Parent terms were removed to keep the most specific GO annotations. Protein domains for each organism were downloaded from the Pfam database [48]. Only distinct domain annotations for each protein were retained. The population means of each group of nodes for each property were determined statistically significantly different or not using the Mann-Whitney-Wilcoxon U test (two sided) as implemented in R. A p-value of less than 0.01 indicates a statistically significant difference. Finally, essential genes were downloaded from the the Online GEne Essentiality (OGEE) Database [49]. Enrichment of essential genes in multi-clustered node was determined according to the Fisher's Exact test as implemented in R.

### Node Removal

We investigated the effect of node removal using Monte Carlo simulations. At each step a random node is removed from the sets of isolated, inter and intra-connector nodes respectively. We then calculate, 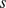, the size (number of nodes) of the largest connected component, over the initial component size. Each step is the average of 100 independent runs.

## Results and Discussion

In this section, the disjoint and overlapping community structures of the PPI networks of rat, *E. coli*, yeast and human are investigated. Hard partitions of the networks are first detected by three different modularity optimisation methods. Based on the hard partitions, each node is classified as either an isolated node or a connector node. Characteristics of the connector nodes are investigated in order to determine whether they possess some topological and/or functional relevance relating to their position in the network. The hard partitions are subsequently transformed to overlapping communities by the proposed mathematical programming method, OverMod. The effect of node removal and the functional significance of the inter and intra-connectors are then explored. Finally, the proposed procedure is discussed in the context of other existing methodologies.

### Detection of Hard Partitions

We employ three of the most well known methods of modularity optimisation to detect hard partitions of the PPI networks: iMod [34], Louvain [27] and QCUT [28]. Each method has been shown to perform well in applications on various sizes of complex networks. In particular, iMod outperformed several other well-known methods on medium to large sized networks, including finding known globally optimal solutions for small sized networks [34] and Louvain, a heuristic method, is known for its low computational cost and high quality results on very large networks [50]. Employing these three methods allows us first to explore the effect of the choice of hard partition on the final results found by OverMod and in turn the stability of the algorithm, and second, to combine the information from them to determine the most robustly multi-clustered proteins. In doing this, we take advantage of the information offered by a range of results from three of the best available methods.

Table 1 gives the value of modularity, the corresponding number of modules and the number of connector nodes found by iMod, Louvain and QCUT for each of the PPI networks. For the rat and *E. coli* networks, iMod finds partitions with marginally larger values of modularity, for the yeast network, Louvain performs best and for the human network QCUT performs best. These differences in modularity are minimal and generally for each network, the methods perform similarly in terms of value of modularity, number of modules and number of connectors. The difference between the three sets of connector nodes produced for each network is quantified by employing the Jaccard index (results not shown). The Jaccard index measures similarity between finite sample sets, and is defined as the number of nodes in the intersection of the sets divided by the number of nodes in the union of the sets. The average of the Jaccard values between pairwise sets of connectors for Rat, *E. coli*, Yeast and Human are 0.78, 0.80, 0.88 and 0.80, respectively. The difference between the sets of connectors found by each method across the networks is relatively small. It is investigated later how these differences are reflected in the output of OverMod, i.e. how stable is the algorithm to perturbations in the input.

**Table 1:** Hard partition summary.

	iMod			Louvain			QCUT		
Network	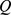	Mod	Con	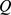	Mod	Con	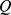	Mod	Con
**Rat**	0.7734	18	54	0.7729	17	56	0.7701	18	54
***E. coli***	0.8537	24	59	0.8523	23	58	0.8522	24	59
**Yeast**	0.7572	20	310	0.7612	22	299	0.7608	21	290
**Human**	0.5382	18	4529	0.5462	19	4372	0.5527	16	4246

Summary of the hard partitions of the rat, *E. coli*, yeast and human PPI networks, showing modularity value (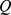), corresponding number of modules (*Mod*) and the number of connectors (*Con*). For each network, the hard partition results appear to be relatively stable. These hard partitions are the input for OverMod.

**Figure 2:**
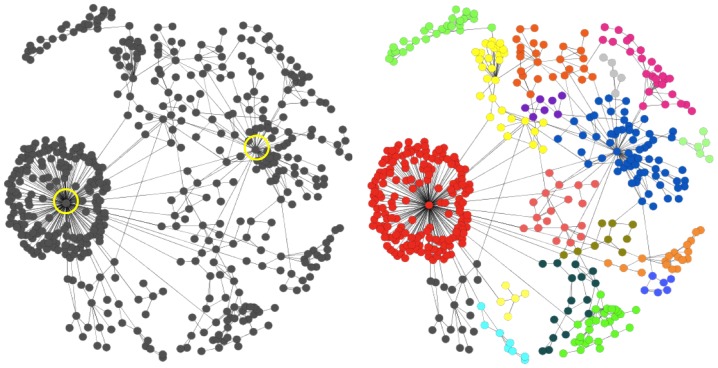
The left hand side of the figure shows the rat PPI network and on the right hand side, each community found by iMod is given an individual colour. Hub nodes (SUMO3 and UBC) are highlighted by yellow circles in the network on the left. Visualisation of the network was done using Cytoscape [82].

Finally, as an illustrative example of the hard-partitioning step, Figure 2 shows the rat PPI network before partitioning, followed by the clustered network, where the modules found by iMod are identified by an individual colour. Hub nodes, UBC (54 interaction partners) and SUMO3 (187 interaction partners), are identified by yellow circles. The hard partition is dominated by one large module (red). It comprises 168 nodes and corresponds to the SUMO3 gene and its surrounding nodes, while the second largest module (blue), comprises the UBC gene and 63 surrounding nodes.

#### Properties of Connector Nodes

We investigate the nature of the two types of nodes that are defined according to the hard partitions: isolated and connector nodes. If connector nodes are interpreted as bridges between functional units, one expects them to exhibit properties that reflect such activity. Here, various topological and functional measures are employed to characterise protein nodes and investigate whether connectors can be distinguished from isolated nodes in terms of these properties.

The following topological properties are employed as measures of a node's structural features. First, node degree is used as it has been shown that complex networks are vulnerable to targeted removal of nodes with a high degree, also known as hubs [51], [52]. Hubs therefore are considered to possess particular topological characteristics that may also link to relevant functional properties. Second, node betweenness is used to indicate the number of shortest paths that traverse a particular node. It has been suggested that nodes of high betweenness usually lie between communities, according to the betweenness clustering method of Girvan and Newman [53], potentially indicating connector properties. Finally, eigenvector centrality is considered, which is a method of computing the centrality of a node based on the centrality values of the nodes that it is connected to.

Where the above properties offer topological measures for illustrating structural importance, we also consider descriptive features based on protein function. We use the number of GO annotations as a measure of functional importance of a node, both in combined form (ALL GO) as well in terms of the individual GO categories (molecular function, MF, biological process, BP and cellular compartment, CC). Additionally, the number of domains that a protein contains are used as measure of its multi-functionality. Finally, the Online GEne Essentiality (OGEE) Database [49] that contains genes that have been tested experimentally for essentiality, is employed here and we investigate whether the connector nodes are enriched for essential proteins.

For all four networks node degree, node betweenness and eigenvector centrality were on average significantly higher for connector nodes when compared to isolated nodes (Table S1 in File S1), thus demonstrating their distinct topological properties. We now reinforce these results by showing that topological features correspond to particular functional properties. First, it has been shown that signalling domains are found more often in intermodular hub proteins, which were also more frequently associated with oncogenesis than their intramodular hub counterparts [54]. Our analysis therefore demonstrates that the high connectivity of these nodes is correlated with their multiple roles and their potential to act as bridges between functional modules.

In the human network, connectors have a significantly higher average number of GO annotations and protein domains (Table S2 in File S1). While in the rat network, this is true for GO terms. For the yeast and *E. coli* networks these effects are less pronounced, perhaps owing to the fact that, as yeast and *E. coli* are unicellular organisms, they have much simpler biochemistry than multicellular species. The difference in organismal complexity may be therefore reflected, in this case, in the significance of GO term enrichment. Additionally, with respect to essentiality properties, we find significant enrichment of essential genes in the connector nodes of *E. coli*, yeast and human (Table S3 in File S1). For the rat network, only a small number of essential genes have been identified, so statistical evaluation was not attempted.

The p-values for each property do not vary greatly between the different hard partitioning methods employed, i.e the overall properties of connector nodes are generally the same regardless of the hard partitions used. The above results indicate then that in general, connector nodes have not only distinct topological properties, but also a wider functional repertoire than isolated proteins. Our next task was to investigate the properties of connector nodes through the detection of overlapping communities (soft partition), which we discuss in the following section.

### Converting to Soft Partitions

We denote the application of OverMod to each hard partition as the following three methods: (i) iMod for the hard partition followed by OverMod, (ii) hard partition by Louvain followed by OverMod and (iii) hard partition by QCUT followed by OverMod. Applying OverMod to the hard partitions results in connector nodes being determined as either inter or intra-connector. Table 2 summarises the number of inter and intra-connectors.

**Table 2:** Soft partition summary: inter and intra-connectors.

	(i)		(ii)		(iii)	
	Inter	Intra	Inter	Intra	Inter	Intra
**Rat**	45	9	48	8	45	9
*E. coli*	51	8	51	7	50	7
**Yeast**	274	36	274	25	260	30
**Human**	4207	322	3952	420	3647	559

Summary of the number of connectors that become inter and intra-connectors in the corresponding soft partitions. For Rat, *E. coli* and Yeast in particular, we see that the number of inter-connectors, i.e. nodes belonging to more than one community, found by each method (i) – (iii) are relatively similar. This stability is reinforced in Figure 4, where we see that the number of common inter-connectors is high for each PPI network.

**Figure 3:**
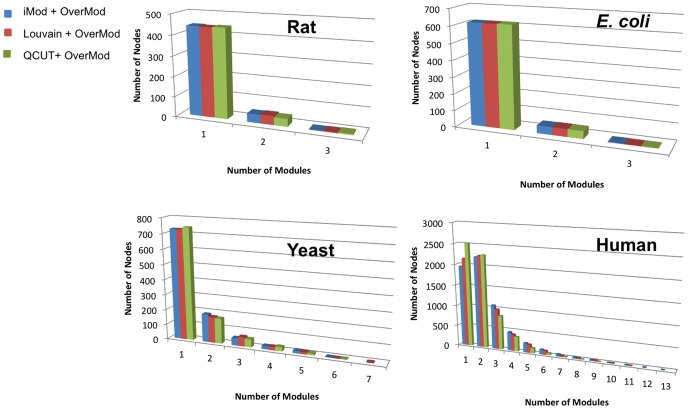
Bar charts showing the number of modules and the corresponding number of proteins for each soft partition for the rat, *E. coli*, yeast and human PPI networks.

Figure 3 shows the number of modules that protein nodes belong to across the reference organisms tested. Nodes belonging to only one module include both isolated and intra-connector nodes. For rat and *E. coli*, the inter-connector nodes belong to at most 3 modules, whereas inter-connector nodes participate in up to 7 and 13 modules in the yeast and human networks respectively.

**Figure 4:**
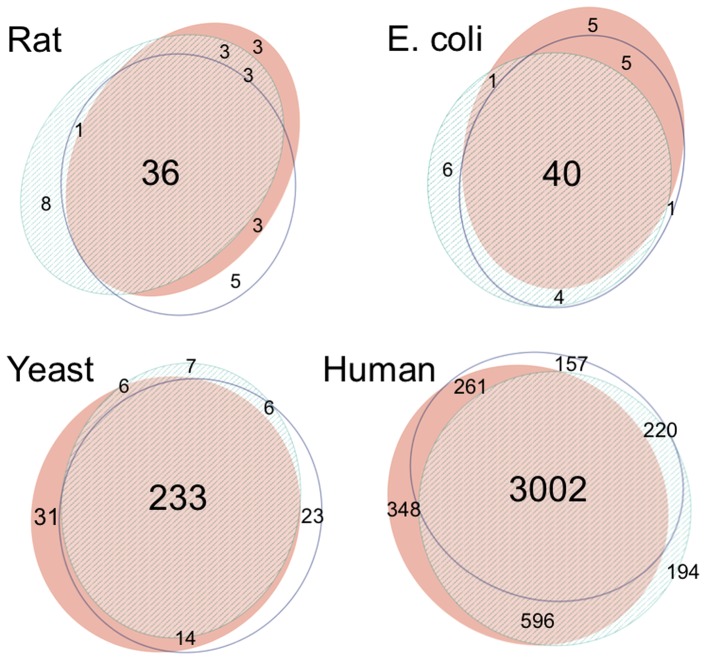
Venn diagrams illustrating the number of common inter-connector proteins across the methods for each network. The pink ellipse represents iMod + OverMod, the green striped ellipse represents Louvain + OverMod and the empty ellipse is QCUT + OverMod.

The stability of OverMod is now investigated, i.e. it is determined how much the output is perturbed by changes to the input. In this case the input is the hard partition found by either iMod, Louvain or QCUT and the output is the classification of connectors as either inter or intra-connectors. Of particular interest is how the sets of inter-connectors vary depending on which hard partition is used as input. The commonality between inter-connector nodes across methods (i) – (iii) is illustrated in Figure 4. Inter-connector proteins found by all three methods were 36 in the rat network, 40 in *E. coli*, 233 in yeast, and 3002 in the human network. The Jaccard index is again employed as a means of quantifying the similarity between the inter-connector sets returned by OverMod for methods (i) – (iii). The Jaccard index has previously been used a measure of cluster stability [55]. For each PPI network, the Jaccard index is calculated for iMod + OverMod vs. Louvain + OverMod, iMod + OverMod vs. QCUT + OverMod and Louvain + OverMod vs. QCUT + OverMod. Results are presented in Table S4 in File S1 and here the average Jaccard index for each network is reported: 0.72, 0.75, 0.85 and 0.74 for the rat, *E. coli*, yeast and human networks, respectively. The similarity between inter-connector sets is relatively stable across all networks. Overall, the results show a good degree of stability regarding individual inter-connector proteins, indicating that OverMod exhibits a good level of robustness to small perturbations in the input.

#### Topological Features and the Effect of Node Removal

Node degree, betweenness and eigenvector centrality of the two types of connector nodes were calculated. Statistical analysis showed a significant difference in node degree for the rat, *E. coli* and human networks, with the intra-connectors having consistently higher values (Table S5 in File S1). For the yeast network, although inter-connectors have a higher average degree than intra-connectors, the difference is not significant. Less consistent results are found for betweenness and eigenvector centrality.

On first inspection, the observation that nodes characterised as intra-connectors by OverMod had higher degree was counter-intuitive, we therefore sought to look more closely at inter-module connections to determine if inter- and intra-connectors can be characterised according to their communication with neighbouring modules. A measure known as participation coefficient is adopted, which measures how uniformly distributed the edges of a node are among the communities of a partition [26]:
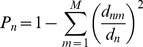
(8)where 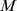 is the number of modules in the partition, 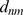 is the number of links node 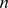 has with nodes in module 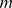 and 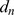 is the degree of node 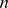. The larger the participation coefficient of a connector, the more evenly distributed its connections are with different modules. We found that for all four networks studied, inter-connectors have a significantly higher average participation coefficient than intra-connectors (Table S6 in File S1), indicating that despite lower node degree on average, inter-connector genes distribute their edges across communities more widely than intra-connectors.

**Figure 5:**
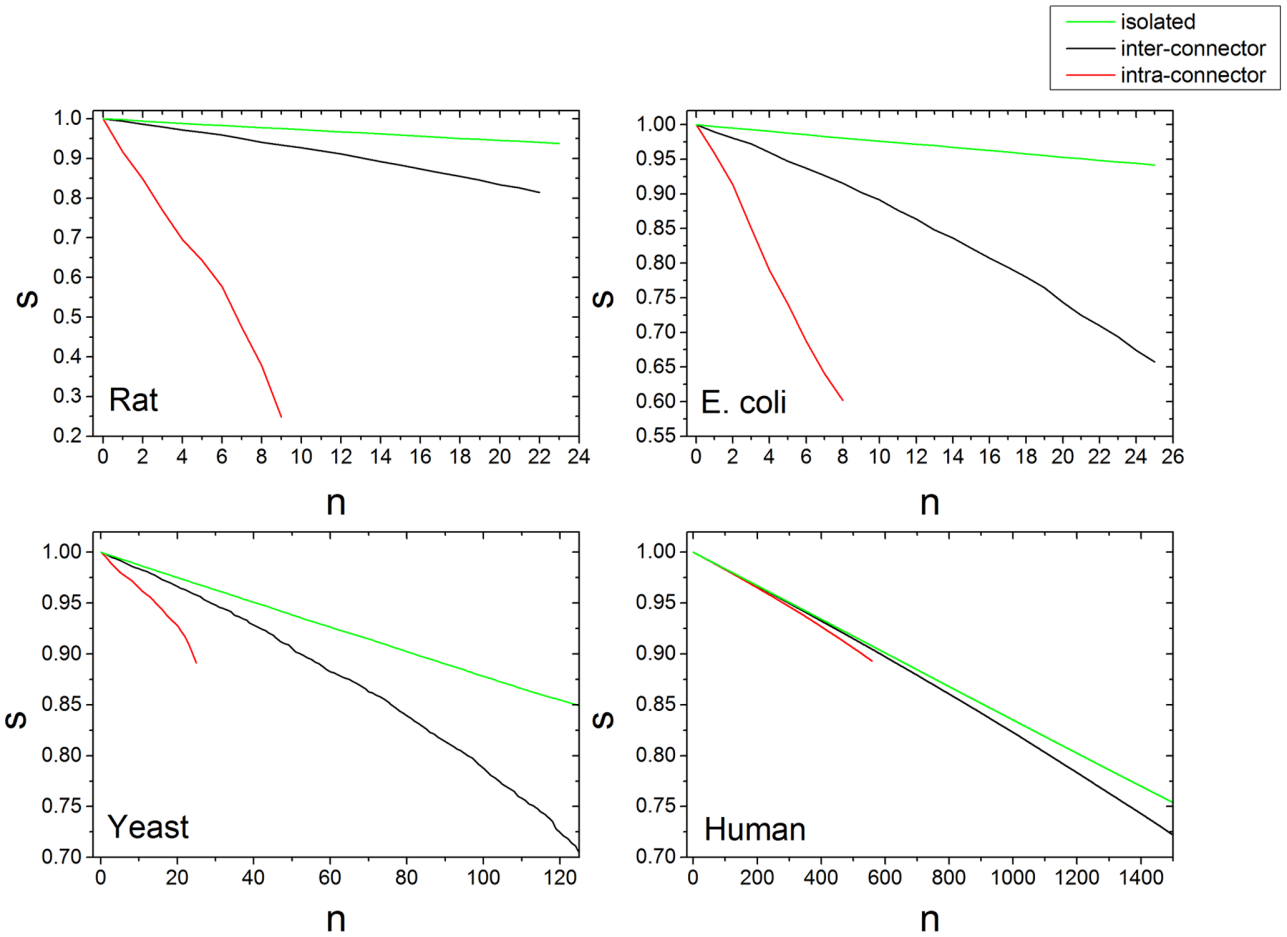
Relative size of largest component 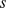 vs number of nodes removed 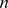 for human, yeast, *E. coli* and rat networks. At each step, a random node is removed from the set of isolated, inter-connector and intra-connector nodes respectively. Results are an average of 100 runs. From each organism, the hard partitioning method which yielded the best Q was selected, namely iMod for the rat and *E. coli* networks, Louvain for yeast and QCUT for human

The topological properties of inter-connector, intra-connector, as well as isolated nodes were also established through simulating the effect of their removal on network integrity. Figure 5 shows the relative size of the largest component 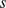 against the number of nodes 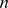 removed from the network. In all cases the removal of isolated nodes has the smallest effect on network structure, in line with our results of centrality of isolated vs connector nodes, where degree and betweenness centrality are always significantly lower in isolated nodes. This illustrates that the isolated nodes are the least important in maintaining network integrity.

Our simulations also show that the removal of intra-connector nodes breaks the network consistently faster than inter-connectors, in accordance with their significantly higher node degree. The effect can also be seen in the yeast and human networks, however it is less pronounced due to the density and size of these networks, i.e. a large portion of the nodes need to be removed in order to reduce the largest component size. Alternatively, in rat and *E. coli* where the effect was more marked is attributed to the fact that the integrity of these smaller networks is largely maintained by a few intra-connectors (Table 2), which for both networks contain the most highly connected nodes in the entire network. These two cases are discussed in more detail below.

The most highly connected node in the rat PPI network (degree 187) corresponds to the SUMO3 gene and the most highly connected node in the *E. coli* networks is the CH60 (GroEL) protein (degree 128). Both are intra-connector nodes despite their large degree and the fact that they interact with nodes in 12 and 9 modules respectively. In other words, there is a large number of possible modules that OverMod could assign them to. We investigate why these two nodes are intra-connectors, when the highest degree nodes in the yeast and human networks are inter-connectors.

We consider the application of OverMod to the hard partitions found by iMod in the following discussion regarding the rat and *E. coli* networks. SUMO3 is not only the node with the highest degree in the rat network, it is also the node with the largest number of possible modules it can be assigned to. However, OverMod, assigns SUMO3 to only one module. On further investigation, we find that only 20 out of 187 connections link to nodes in other modules, indicating (i) a strong connection to the module where it was assigned during the hard partition stage and (ii) a rather low participation coefficient of 0.2. Bearing in mind that OverMod simultaneously optimises the modularity of all modules in the soft partition by either assigning the connector nodes to one or multiple modules, we calculate the modularity for each of the 12 possible modules, with SUMO3 present and absent. It is found that SUMO3 decreases the modularity of all modules other than its own. Therefore, to optimise the total modularity across the soft partition, OverMod assigns SUMO3 to its own module only.

Similarly, in the *E. coli* network, the CH60 (GroEL) protein, also has the highest number of possible modules it can be assigned to. However, of its 128 interactions, only 12 are among the 9 neighbouring modules, corresponding to a participation coefficient of 0.18. Again, CH60 was found to decrease the modularity of all neighbouring modules and only increase the modularity of its own module, despite its high centrality and potential to belong to many modules. These two cases illustrate that high degree is not necessarily the principal driving force when OverMod allocates a connector node to more than one module. In fact, these two examples are representative of high degree connector nodes that become intra-connectors and thus why so many high degree connectors remain in a single module.

We compare these cases with the most highly connected nodes in the yeast and human networks, which are both multi-clustered by OverMod. These have degrees 127 and 182 respectively. However, the difference is their much higher participation coefficient, 0.5 and 0.7 respectively. Therefore these high centrality nodes have a more even distribution of edges, whereas the corresponding nodes in the rat and *E. coli* have most edges concentrated in their original module. It is worth noting that if one calculated the average degree of the intra-connectors for the rat and *E. coli* networks without SUMO3 and CH60 respectively, the intra-connectors would still show a significantly higher average degree than the inter-connectors (with no significant difference for betweenness and eigenvector centrality as before). Similarly, inter-connectors continue to have a significantly higher participation coefficient than intra-connectors.

Overall, these results show that OverMod reveals the structural significance of a portion of the connector nodes, i.e. those with high connectivity which end up assigned to only one of their possible modules. While the majority of connectors maximise modularity by becoming inter-connectors (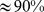 in most cases), a small number of highly connected nodes optimise modularity by remaining in their original module. Thus our method discovers a small subset of intra-connectors that are topologically important for the integrity of the network. OverMod also identifies the set of inter-connectors which may generally have a lower degree, but exhibit a higher participation coefficient, signifying that their edges are more evenly distributed among various modules. We now investigate the functional properties of inter- and intra-connectors and in particular we look into the biological evidence that corresponds to the association of multi-clustered nodes with multiple functional units.

#### Functional Comparison of Inter and Intra-connector Proteins

A PPI network is a collection of interactions that take place across time and space, no matter whether they happen simultaneously or not, or whether they are exclusive or not. Therefore, a PPI network may not capture a precise representation of protein interactions *in vivo*. While high centrality has been traditionally correlated with essentiality for survival [56]–[58], we investigate below whether this is consistently the case by comparing the most robust inter-connectors and intra-connectors with the highest connectivity.

First, we determine which nodes are considered as ‘strong’ inter-connectors. For the rat and *E. coli* networks, since all inter-connector proteins belong to either 2 or 3 modules, we consider proteins belonging to 3 modules as strong inter-connectors. For the yeast and human networks, the number of modules that inter-connector nodes belong to is higher and so strong inter-connectors are defined by finding the range of number of modules to which the top ten strongest inter-connectors are assigned. For example, for the yeast network, the top ten strongest inter-connectors for iMod+OverMod belong to between 5 and 6 modules. However, there are actually 19 nodes that belong to 5 and 6 modules and therefore we consider all of them as strong inter-connector proteins. It follows that any inter-connector defined as strong by two or more methods is described as being robust (Table 3). We now examine the biological functionality of robust inter-connector proteins and high degree intra-connector proteins with high and low inter-modular degree (shown in tables 3 and 4 respectively).

**Table 3:** Robust inter-connectors.

**Rat**	MDM2, BRDT, *HSP90AA1*, *SUMO1*
***E. coli***	EF-Tu1, MutL, *DNA-Pol III*
**Yeast**	RPL31A, NOP1, RPS4A, RPL7B, RPS7A, RPS8A-B, RPS5, RPS11A-B, XRN1, RPS22A, *RPS13*,*RPS9B*, *SRO9*, *PRP43*,*RPL8B*, *CBF5*
**Human**	GBLP, CSK21, HS90A, ANDR, CDC2, *RAF1*, *CTNB1*, *RB*, *A4*, *NPM*, *UBIQ*, *1433Z*

Connector proteins that were found to be strong inter-connectors by OverMod when applied to: (i) all three hard partitions, (ii) two out of three hard partitions (italics).

**Table 4:** Intra-connector proteins.

**Rat**	UBC, SUMO3, **UBC9, NTRK1, TBA1A, HSP74**
***E. coli***	CH60, DNAK, **HLDD, KPRS, ODP1, RPE, RS2, SYP**
**Yeast**	**CLP1, CKA2, CKB1-2, YEF3, FKS1, VID24, GCD11, NAP1, PAP1, PCF11, PTI1, REF2, MRP4, MRP7, RNA14, RSM19M RSMM22, RSM23M, MRP51, MRPS9, MRPS18, SWD2, YSH1**
**Human**	TGFR1, EF1G, ATN1, MDFI, PLS1, TRIP6, CACO2, KR412, AP2M1, MCM7, PABP1, ARI2, FHL3

Proteins with inter-modular degree that is (i) greater than 5 and (ii) equal to or less than 5 (bold). Only the former are shown for human, as the number of low inter-modular degree proteins is too large in this network.

We investigate connectors by considering cases of proteins in the literature with significant functionality. CH60 (GroEL) is a molecular chaperone and belongs to a group of proteins that assist in the folding, translocation and assembly of proteins in the cell [59] and are the subject of significant research interest. Another example is Ubiquitin, which is conjugated to target proteins via an isopeptide bond either as a monomer (monoubiquitin - UBQ), a polymer linked via different Lys residues of the ubiquitin (polyubiquitin chains - UBC). The linkage type of the ubiquitin chain determines whether a modified protein is degraded by the proteasome or serves to attract proteins to initiate signalling cascades or be internalised [60]. UBC is assigned as intra while UBQ as inter-connector. The various types of Ub modifications are linked to distinct physiological functions in cells. UBQ, for example, regulates DNA repair and receptor endocytosis, whereas lysine 48-linked Ub chains label proteins for proteasomal degradation [61]. Since less is known about the functionality of UBC chains than the UBQ monomer, it is possible that UBQ rather than UBC becomes an inter-connector because of its associated multi-functionality, as the molecular mechanisms involving specificity in UBC chain synthesis and recognition are still incompletely understood [62] and thus less information about UBC interactions exists in PPI databases.

Genes in the RPS family encode approximately 80 different ribosomal proteins, which in conjunction with rRNA make up the ribosomal subunits. RPS4A, RPS5, RPS8A etc. are such proteins. RPS5 is a component of the small ribosomal subunit. Mature ribosomes consist of a small (40S) and a large (60S) subunit. Because the ribosome is such a vital component of the translational machinery and therefore of all cellular life, ribosomal proteins (RPs) have been highly conserved throughout evolution [63], [64]. The RSM and MRP genes encode proteins of the 37S small subunit of mature mitochondrial ribosomes [65]. Surprisingly, only a minority of MRPs that have been characterised show significant sequence similarities to known ribosomal proteins from other sources [66]. With respect to our analysis, ribosomes in the cytosol are found to be inter-connectors while mitochondrial ribosomes are intra-connector proteins. We hypothesise that this is the case because cytosol ribosomes have a broader functionality, while mitochondrial ribosomes participate in a more limited spectrum of functions, since their main role is to synthesise proteins of these organelles.

The TGF-beta type I receptor is a transmembrane kinase which transduces TGF signalling from the cell surface to the cytoplasm and thus regulates a plethora of physiological and pathological processes [67]–[70]. Although TGF-beta is important in regulating crucial cellular activities, the full mechanism behind the suggested activation pathways is not yet well understood. Some of the known activating pathways are cell or tissue-specific, while some are seen in multiple cell types and tissues [71], [72]. TGF beta receptor was the intra-connector node with the highest inter-modular degree (equal to 12), so it links to twelve different modules, yet remains assigned in its original module. Because it acts as a signalling molecule activating many pathways but not directly participating in the biochemical processes (i.e. further interacting with molecules in the pathway), it is possible that while it's a very central protein, it remains assigned to a particular module rather than to multiple modules.

GBLP (Guanine nucleotide-binding protein), a component of the small (40S) ribosomal subunit, interacts with a wide variety of proteins and is involved in the recruitment, assembly and regulation of a variety of signalling molecules. CSK21 is a kinase complex that phosphorylates a large number of substrates containing acidic residues C-terminal to the phosphorylated serine or threonine and regulates numerous cellular processes, such as cell cycle and apoptosis [73]. CDC2 encodes the protein CDK1 (Cyclin dependent kinase 1), which is a highly conserved cell cycle protein that forms complexes that phosphorylate a variety of target substrates, leading to cell cycle progression [74]. RAF1 is a proto-oncogene which encodes the c-RAF enzyme. It functions as a switch determining cell fate decisions by acting as a regulatory link between the membrane-associated Ras GTPases and the MAPK/ERK cascade [75], [76]. Mouse double minute 2 homolog (MDM2) is an important negative regulator of the p53 tumor suppressor, functioning both as an E3 ubiquitin ligase that binds to the p53 tumor suppressor and an inhibitor of p53 transcriptional activation [77]. Cell cycle and cancer related-proteins are thus often candidates for inter-connector molecules, as they regulate key processes (such as cell cycle, proliferation and apoptosis) that are interlinked for the cell's survival and reproduction.

Therefore, we see some similarity in the role of connector nodes, but also many differences. We have discovered that heat shock proteins in the rat network can be classified as both inter and intra-connector (HSP74 and HSP90, Tables 3 and 4). In general, intra-connectors do not participate directly in biochemical pathways, but may act as signalling molecules providing multiple connections between the modules (e.g TGF-beta receptor). Cytosol ribosomal proteins in yeast are consistently classified as inter-connectors in contrast to mitochondrial ribosomal proteins which end up in a single module. Overall, many of the robust inter-connectors exhibit the following characteristics:

1. Proteins which are major regulators of the cell cycle and therefore proliferation and apoptosis
2. Proto-oncogenes, oncogenes and regulators of tumour suppressors
3. Proteins which markedly affect cell growth
4. Ribosomal proteins which are essential for the survival and function of the organism and other highly conserved proteins

In a PPI network a protein usually has multiple copies, each acting as a specific molecular entity. These copies may interact with different groups of molecules in the cell [78]. A PPI network would then contain a single vertex that actually represents a collection of that kind of protein, rather than individual protein copies [79]. This is why a hub vertex can bind to hundreds of interactors in a PPI network, while this is unrealistic in biological cells. Assuming that modules correspond to functional units, with this method we suggest candidate proteins of multiple distinct roles (inter-connectors), by their participation in different distinct modules, rather than hubs which are regulators of highly specific pathways.

### Related Methodologies

The overlapping community structure problem has been subject to multiple interpretations due to lack of formalisation of the underlying problem statement. The great variation in existing methodology is reflected in the results, which can even be seen on a small network such as the benchmark Zachary karate network [36]. Here we present a comparative analysis of OverMod with various alternative overlapping community structure detection methods from the literature, namely, CFinder [4], OSLOM [5], OCG [6], ClusterONE [7], GANXiSw [8], MOFinder [9], RSRGM [11] and R-MCL [15]. The performance of the above methods will be evaluated on a series of synthetic networks and the PPI networks described previously.

First, we note Wang et al. [12] have proposed a similar method to OverMod involving the two stage optimisation of modularity. Here we have addressed the problem more comprehensively and in a more rigorous mathematical manner. By employing mathematical programming and optimising the sum of modularity across all modules in the soft partition, we avoid sequence dependent results, as well as offering the possibility for a connector node to leave its original module in the hard partition. Furthermore, in our evaluation, we focus on the nature of multi-clustered nodes in the context of PPI networks, instead of the functional cohesion of modules. Since, a method implementation is not publicly available, we do not provide results.

A series of synthetic networks of the type described in [80] was generated. Each network comprises 500 nodes, with average degree equal to 10 and with either 75, 150 or 250 (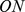) multi-clustered nodes belonging to either 4 or 8 (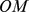) modules. Therefore there are 6 sets of synthetic networks: (i) 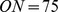, 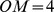, (ii) 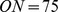, 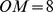, (iii) 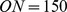, 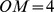, (iv) 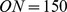, 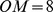, (v) 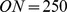, 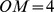 and (vi) 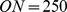, 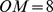. For (i)–(vi), 10 networks were generated for each of the following mixing parameters (the fraction of all links that lie between modules): 0.05, 0.1, 0.2 and 0.3. OverMod, CFinder, OSLOM, OCG, ClusterONE, GANXiSw, MOFinder and R-MCL were applied to the synthetic networks. Note that RSRGM was not evaluated on the synthetic networks as an upper bound for the number of modules is required to be selected in advance. Louvain was used to find the hard partition for OverMod, levels 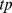 and 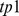 of the OSLOM results were considered, for GANXiSw, 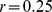 and for CFinder, 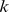 was set to 3, 4 and 5. Otherwise, all default parameters were selected.

**Figure 6:**
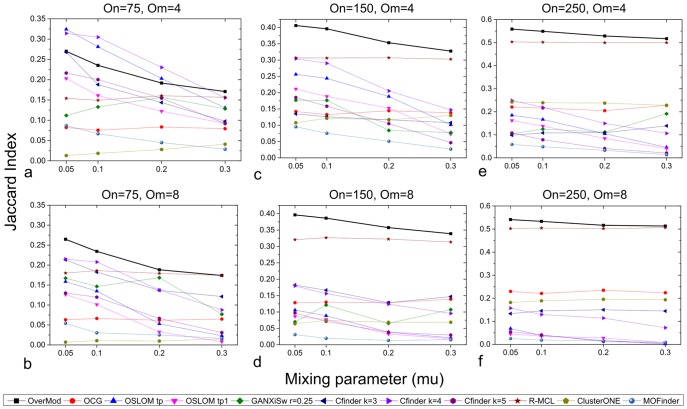
Method comparison on synthetic networks. The average Jaccard index between the sets of ‘known’ multi-clustered nodes and those predicted to be multi-clustered by OverMod and other overlapping community structure detection methods from the literature.

The aim of OverMod is to identify multi-clustered nodes that adopt the important role of bridging multiple modules. OverMod and the alternative methods are therefore evaluated based on their ability to identify those multi-clustered in synthetic networks. The Jaccard index is employed here to quantify the similarity between the set of ‘known’ multi-clustered nodes (as given by the synthetic network generating software) and those predicted by the clustering methods being evaluated. Figure 6 shows the average Jaccard index against the mixing parameter for (i)–(iv) above. For (i), OSLOM 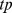 and CFinder 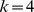 identify the multi-clustered nodes more accurately than OverMod, however for (ii) to (vi) OverMod performs best. Note that there are no results for GANXiSw for (vi) as for many networks the method only found one module. Overall, OverMod identifies the known multi-clustered nodes well for a range of synthetic networks with varying connectivity properties.

Synthetic networks offer a means of benchmarking community structure detection methods, however, determining parameters such as average degree, 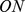 and 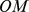, do not represent real life complex networks. As such, each method, including RSRGM, is now applied to the PPI networks analysed previously. For RSRGM the upper bound on the number of modules was taken to be the average of the results for all other methods for each network. Table 5 summarises the results in terms of number of modules (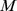), number of nodes that are assigned at least one community (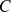), number of nodes that are multi-clustered (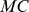) and the maximum number of modules the multi-clustered nodes belong to (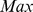). Clearly, the results vary to a large degree for all of these factors. In particular, the range of number of modules in each soft partition for each network is vast, e.g. for the rat network partitions have from between 3 and 328 modules. Note also that for the rat PPI network, OSLOM finds only 1 multi-clustered protein, CFinder (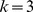) finds 4 multi-clustered proteins but only 74 our of 487 nodes are assigned at least one module and CFinder (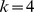) clusters only 12 nodes with no multi-clustered nodes. Similarly for the *E. coli* network, OSLOM finds only 1 multi-clustered node. Therefore, for some applications, these methods may not offer satisfactory or relevant solutions.

**Table 5:** Method comparison on the PPI networks.

	Rat				*E. coli*				Yeast				Human			
	M	C	MC	Max	M	C	MC	Max	M	C	MC	Max	M	C	MC	Max
CFinder 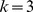	20	74	4	4	17	97	5	2	N/A	N/A	N/A	N/A	364	2847	477	6
CFinder 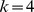	3	12	0	N/A	6	37	3	2	N/A	N/A	N/A	N/A	126	808	173	9
CFinder 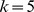	N/A	N/A	N/A	N/A	8	16	0	N/A	N/A	N/A	N/A	N/A	31	267	28	5
OSLOM 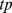	8	487	1	2	9	668	1	2	57	1002	65	3	157	6160	657	6
OSLOM 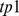	N/A	N/A	N/A	N/A	N/A	N/A	N/A	N/A	16	1002	216	3	48	6160	1048	5
OCG	328	487	86	172	401	668	50	125	252	1002	585	41	393	6160	2104	53
ClusterONE	27	91	11	3	38	140	30	3	115	910	172	4	572	2202	338	4
GANXiSw 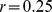	36	487	22	2	42	668	16	2	58	1002	25	2	302	6160	1120	3
MOFinder	12	37	12	4	13	52	13	4	143	695	316	8	95	338	86	6
RSRGM	43	425	55	3	64	572	60	3	78	837	112	4	651	4688	1028	4
R-MCL	205	476	267	22	360	660	503	19	222	957	588	12	5297	6160	6112	28

Summary of results found by applying the methods from the literature to the four PPI networks in terms of number of modules (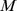), number of nodes that are assigned at least one community (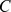), number of nodes that are multi-clustered (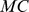) and the maximum number of modules the multi-clustered nodes belong to (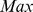). The results highlight the great variation between each method. Please note that CFinder did not complete within 24 hours for the yeast PPI network, there was no 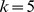 result for CFinder for the rat PPI network and for the rat and *E. coli* networks, OSLOM did not produce a partition for level 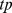.

The Jaccard index is employed to carry out a pair-wise comparison between the sets of multi-clustered proteins found by all methods for each of the four PPI networks (results not shown). For the rat PPI network, the two methods with the most similar set of multi-clustered nodes are OCG and R-MCL. Furthermore, these are the two methods with results that are most similar to OverMod. The same is true for the *E. coli* network. For the Yeast network, MOFinder, closely followed by OCG and R-MCL, generate sets of multi-clustered nodes most similar to those found by OverMod. For the human network, R-MCL finds the most similar multi-clustered nodes to OverMod. Furthermore, OCG and R-MCL multi-cluster all of the robust inter-connectors (Table 3) for the Rat, *E. coli* and Human networks, while the same methods, and additionally MOFinder, multi-cluster all of the robust inter-connectors for the Yeast network.

Evaluating method performance on the PPI networks is difficult. Unlike the synthetic networks, a benchmark of known multi-clustered nodes is not available. Each method's approach varies to such a large degree and for many of the above methods, choice of parameter values can greatly affect the final results, with often no way of determining the ‘correct’ values. Furthermore, as our results show, and has also previously been reported [12], CFinder generally leaves a large portion of the network un-clustered. Similarly, ClusterONE, MOFinder, RSRGM and R-MCL do not always assign each node to at least one module. Each of these factors makes a fair comparison very difficult. Ultimately, one must choose a method that is suited to their application and user requirements. Based on the assumption that modularity optimisation is meaningful in biological networks, we have chosen our proposed two-stage approach and in light of the results presented in the previous sections, we believe OverMod to be a reasonable and successful approach to finding overlapping communities and in particular identifying ‘important’ nodes in PPI networks.

## Conclusions

In this work, a two-stage procedure for identifying overlapping community structure is outlined. Stage one involves detecting disjoint communities of a network using existing methodologies. In stage two, we propose, OverMod, a novel mixed integer non-linear programming (MINLP) model to convert disjoint to overlapping network communities. We extend the use of modularity optimisation and mathematical programming in community detection and present a thorough investigation into the relevance of this methodology in PPI networks. Connector proteins exhibited a range of topological and functional properties indicative of their role in these networks, thus demonstrating their suitability as candidate nodes for multiple module membership. OverMod was then shown to identify two types of connector nodes: inter and intra-connector, each with their own distinguishing topological features. In general, intra-connectors have a higher node degree than inter-connectors, their removal breaks down the network faster, while inter-connectors exhibit a larger dispersion of their connections across modules, thereby acquiring a higher average participation coefficient. Further investigation suggested characteristics that differentiated these nodes in terms of functionality.

Through the above discussion of our results and comparison to other methods, the comparative advantages of the two-stage procedure become apparent. In particular, OverMod can be applied to any hard partitioning method that is deemed suitable to the problem. Owing to the nature of the mathematical programming framework used, modelling can be flexible enough to allow additional constraints and parameters to be easily implemented, as relevant according to user requirements. Prior knowledge on a particular system can be incorporated, for example in the form of nodes with similar functional annotations that may be constrained to be allocated in the same community. Modelling and solution procedure enhancements can also be investigated in order to improve the efficiency of OverMod (e.g. symmetry breaking constraints [31], column generation techniques [32] solution post processing [81]).

Overall, the development of overlapping community detection procedures has the potential to uncover the principles of communication across distinct functional modules through the investigation of nodes which connect cellular processes, providing a greater understanding of system properties. OverMod identifies two types of connector proteins that may play different but central roles in linking distinct processes, distinguishing them from single hubs that may connect a large number of possibly functionally homogeneous interacting proteins.

We have demonstrated the potential of inter-connectors through their participation in different modules, providing a reasonable interpretation of proteins with multiple interactors in a PPI network, since this is unrealistic in biological cells. Many of these proteins are major regulators of proliferation and apoptosis, including oncogenes and regulators of tumour suppressors. The application of our method in disease networks may therefore be relevant in prioritising OMICs results, especially when looking for disease biomarkers, suggesting potential drug targets and regulators of disease related pathways. These results demonstrate the potential of the proposed method in future functional genomics applications and especially in discovering important proteins in less well-characterised systems.

## Supporting Information

File S1 **Supporting tables.** Table S1, The significance values for the comparison of isolated nodes with connector nodes (topological features). Table S2, The significance values for the comparison of isolated nodes with connector nodes (functional features). Table S3, Essentiality results summary, where p-values less than 0.01 indicate that connector nodes in the corresponding organism are significantly enriched for essential genes. Table S4, The Jaccard index for each pair-wise comparison of sets of inter-connector nodes. Table S5, Summary of the significance values for the comparison between inter and intra-connectors based on topological measures. Table S6, Average participation coefficient for inter and intra-connectors with corresponding significance values.
(PDF)

## References

[1] Lewis A, Jones N, Porter M, Deane C (2010) The function of communities in protein interaction networks at multiple scales. BMC Systems Biology 4: 100. 20649971 10.1186/1752-0509-4-100 PMC2917431

[2] Voevodski K, Teng SH, Xia Y (2009) Finding local communities in protein networks. BMC Bioinformatics 10: 297. 19765306 10.1186/1471-2105-10-297 PMC2755114

[3] Kühner S, van Noort V, Betts MJ, Leo-Macias A, Batisse C, et al (2009) Proteome Organization in a Genome-Reduced Bacterium. Science 326: 1235–1240. 19965468 10.1126/science.1176343

[4] Palla G, Dernyi I, Farkas I, Vicsek T (2005) Uncovering the overlapping community structure of complex networks in nature and society. Nature 435: 814–818. 15944704 10.1038/nature03607

[5] Lancichinetti A, Radicchi F, Ramasco JJ, Fortunato S (2011) Finding Statistically Significant Communities in Networks. PLoS ONE 6. 10.1371/journal.pone.0018961 PMC3084717 21559480

[6] Becker E, Robisson B, Chapple CE, Guénoche A, Brun C (2012) Multifunctional proteins revealed by overlapping clustering in protein interaction network. Bioinformatics 28: 84–90. 22080466 10.1093/bioinformatics/btr621 PMC3244771

[7] Nepusz T, Yu H, Paccanaro A (2012) Detecting overlapping protein complexes in protein-protein interaction networks. Nature Methods 9: 471–472. 22426491 10.1038/nmeth.1938 PMC3543700

[8] Xie J, Szymanski BK (2012) Towards linear time overlapping community detection in social networks. CoRR abs/1202.2465.

[9] Yu Q, Li GHH, Huang JFF (2012) MOfinder: a novel algorithm for detecting overlapping modules from protein-protein interaction network. Journal of biomedicine & biotechnology 2012: 103702. 22500072 10.1155/2012/103702 PMC3303734

[10] Dhillon IS, Guan Y, Kulis B (2007) Weighted graph cuts without eigenvectors a multilevel approach. IEEE Trans Pattern Anal Mach Intell 29: 1944–1957. 17848776 10.1109/TPAMI.2007.1115

[11] Zhang RC, Lin Y, Yue M, Li Q, Zhang XF, et al (2012) Exploring overlapping functional units with various structure in protein interaction networks. PLoS One 7. 10.1371/journal.pone.0043092 PMC3423443 22916212

[12] Wang X, Li L, Cheng Y (2012) An overlapping module identification method in protein-protein interaction networks. BMC Bioinformatics 13: S4. 10.1186/1471-2105-13-S7-S4 PMC3348045 22595001

[13] Asur S, Ucar D, Parthasarathy S (2007) An ensemble framework for clustering proteinprotein interaction networks. Bioinformatics 23: i29–i40. 17646309 10.1093/bioinformatics/btm212

[14] Ucar D, Asur S, Catalyurek U, Parthasarathy S (2006) Improving functional modularity in protein-protein interactions graphs using hub-induced subgraphs. In: Proceedings of the 10th European Conference on Principle and Practice of Knowledge Discovery in Databases. Berlin, Heidelberg: Springer-Verlag, PKDD'06, pp. 371–382.

[15] Shih YK, Parthasarathy S (2012) Identifying functional modules in interaction networks through overlapping markov clustering. Bioinformatics 28: i473–i479. 22962469 10.1093/bioinformatics/bts370 PMC3436797

[16] Liu G, Wong L, Chua HN (2009) Complex discovery from weighted PPI networks. Bioinformatics 25: 1891–1897. 19435747 10.1093/bioinformatics/btp311

[17] Russ AP, Lampel S (2005) The druggable genome: an update. Drug Discovery Today 10: 1607–1610. 16376820 10.1016/S1359-6446(05)03666-4

[18] Shen HW, Cheng XQ, Guo JF (2009) Quantifying and identifying the overlapping community structure in networks. Journal of Statistical Mechanics: Theory and Experiment 2009: P07042.

[19] Wu K, Taki Y, Sato K, Sassa Y, Inoue K, et al (2011) The overlapping community structure of structural brain network in young healthy individuals. PLoS ONE 6: e19608. 21573111 10.1371/journal.pone.0019608 PMC3089616

[20] Salath M, Jones JH (2010) Dynamics and control of diseases in networks with community structure. PLoS Comput Biol 6: e1000736. 20386735 10.1371/journal.pcbi.1000736 PMC2851561

[21] Newman MEJ, Girvan M (2004) Finding and evaluating community structure in networks. Physical Review E 69: 026113. 10.1103/PhysRevE.69.026113 14995526

[22] Chen J, Yuan B (2006) Detecting functional modules in the yeast proteinprotein interaction network. Bioinformatics 22: 2283–2290. 16837529 10.1093/bioinformatics/btl370

[23] Zinman G, Zhong S, Bar-Joseph Z (2011) Biological interaction networks are conserved at the module level. BMC Systems Biology 5: 134. 21861884 10.1186/1752-0509-5-134 PMC3212960

[24] Lee J, Gross SP (2013) Improved network community structure improves function prediction. 10.1038/srep02197 PMC3711050 23852097

[25] Newman MEJ (2004) Fast algorithm for detecting community structure in networks. Physical Review E 69: 066133. 10.1103/PhysRevE.69.066133 15244693

[26] Guimera R, Amaral L (2005) Functional cartography of complex metabolic networks. Nature 433: 895–900. 15729348 10.1038/nature03288 PMC2175124

[27] Blondel VD, Guillaume JL, Lambiotte R, Lefebvre E (2008) Fast unfolding of communities in large networks. Journal of Statistical Mechanics: Theory and Experiment 2008: P10008.

[28] Ruan J, Zhang W (2008) Identifying network communities with a high resolution. Physical Review E 77: 1–12. 10.1103/PhysRevE.77.016104 18351912

[29] Medus A, Acuna G, Dorso C (2005) Detection of community structures in networks via global optimization. Physica A: Statistical Mechanics and its Applications 358: 593–604.

[30] Wakita K, Tsurumi T (2007) Finding community structure in mega-scale social networks: [extended abstract]. In: Proceedings of the 16th international conference on World Wide Web. New York, NY, USA: ACM, WWW '07, pp. 1275–1276.

[31] Xu G, Tsoka S, Papageorgiou LG (2007) Finding community structures in complex networks using mixed integer optimisation. The European Physical Journal B 60: 231–239.

[32] Aloise D, Cafieri S, Caporossi G, Hansen P, Perron S, et al (2010) Column generation algorithms for exact modularity maximization in networks. Phys Rev E 82: 046112. 10.1103/PhysRevE.82.046112 21230350

[33] Agarwal G, Kempe D (2008) Modularity-maximizing graph communities via mathematical programming. Eur Phys J B 66: 409–418.

[34] Xu G, Bennett L, Papageorgiou LG, Tsoka S (2010) Module detection in complex networks using integer optimisation. Algorithms for Molecular Biology 5: 36. 21073720 10.1186/1748-7188-5-36 PMC2993711

[35] Cafieri S, Hansen P, Liberti L (2011) Locally optimal heuristic for modularity maximization of networks. Phys Rev E 83: 056105. 10.1103/PhysRevE.83.056105 21728603

[36] Bennett L, Liu S, Papageorgiou LG, Tsoka S (2012) Detection of disjoint and overlapping modules in weighted complex networks. Advances in Complex Systems 15: 11500.

[37] Aloise D, Caporossi G, Hansen P, Liberti L, Perron S, et al. (2013). Modularity maximization in networks by variable neighborhood search. Bader, David A. (ed.) et al., Graph partitioning and graph clustering. Proceedings of the 10th DIMACS implementation challenge workshop, Atlanta, GA, USA, February 13–14, 2012. Providence, RI: American Mathematical Society (AMS). Contemporary Mathematics 588, 113–127 (2013). doi:10.1090/conm/588.

[38] Rosenthal R (2008) GAMS - A user's guide. Washington D.C., USA: GAMS Development Corporation.

[39] Stark C, Breitkreutz BJ, Reguly T, Boucher L, Breitkreutz A, et al (2006) Biogrid: a general repository for interaction datasets. Nucleic Acids Research 34: D535–D539. 16381927 10.1093/nar/gkj109 PMC1347471

[40] Hermjakob H, Montecchi-Palazzi L, Lewington C, Mudali S, Kerrien S, et al (2004) IntAct: an open source molecular interaction database. Nucleic acids research 32. 10.1093/nar/gkh052 PMC308786 14681455

[41] Collins SR, Kemmeren P, Zhao XC, Greenblatt JF, Spencer F, et al (March 2007) Toward a comprehensive atlas of the physical interactome of saccharomyces cerevisiae. Molecular and Cellular Proteomics 6: 439–450. 17200106 10.1074/mcp.M600381-MCP200

[42] Csardi G, Nepusz T (2006) The igraph software package for complex network research. InterJournal Complex Systems: 1695.

[43] R Core Team (2013) R: A Language and Environment for Statistical Computing. R Foundation for Statistical Computing, Vienna, Austria. Available: http://www.R-project.org.

[44] Dwinell MR, Worthey EA, Shimoyama M, Bakir-Gungor B, DePons J, et al (2009) The Rat Genome Database 2009: variation, ontologies and pathways. Nucleic Acids Research 37: D744–D749. 18996890 10.1093/nar/gkn842 PMC2686558

[45] Ashburner M, Ball CA, Blake JA, Botstein D, Butler H, et al (2000) Gene ontology: tool for the unification of biology. The Gene Ontology Consortium. Nature genetics 25: 25–29. 10802651 10.1038/75556 PMC3037419

[46] Cherry JM, Hong EL, Amundsen C, Balakrishnan R, Binkley G, et al. (2012) Saccharomyces Genome Database: the genomics resource of budding yeast. Nucleic acids research 40. 10.1093/nar/gkr1029 PMC3245034 22110037

[47] Dimmer EC, Huntley RP, Alam-Faruque Y, Sawford T, O'Donovan C, et al (2012) The UniProt-GO Annotation database in 2011. Nucleic acids research 40: D565–D570. 22123736 10.1093/nar/gkr1048 PMC3245010

[48] Punta M, Coggill PC, Eberhardt RY, Mistry J, Tate J, et al (2012) The pfam protein families database. Nucleic Acids Research 40: D290–D301. 22127870 10.1093/nar/gkr1065 PMC3245129

[49] Chen WHH, Minguez P, Lercher MJ, Bork P (2012) OGEE: an online gene essentiality database. Nucleic acids research 40: D901–D906. 22075992 10.1093/nar/gkr986 PMC3245054

[50] Newman MEJ (2011) Communities, modules and large-scale structure in networks. Nature Physics 8: 25–31.

[51] Cohen R, Erez K, ben Avraham D, Havlin S (2001) Breakdown of the internet under intentional attack. Physical Review Letters 86: 3682–3685. 11328053 10.1103/PhysRevLett.86.3682

[52] Dartnell L, Simeonidis E, Hubank M, Tsoka S, Bogle IDL, et al (2005) Robustness of the p53 network and biological hackers. FEBS Letters 579: 3037–3042. 15896791 10.1016/j.febslet.2005.03.101

[53] Girvan M, Newman MEJ (2002) Community structure in social and biological networks. Proceedings of the National Academy of Sciences 99: 7821–7826. 10.1073/pnas.122653799 PMC122977 12060727

[54] Taylor IW, Linding R, Warde-Farley D, Liu Y, Pesquita C, et al (2009) Dynamic modularity in protein interaction networks predicts breast cancer outcome. Nature Biotechnology 27: 199–204. 10.1038/nbt.1522 19182785

[55] Hennig C (2007) Cluster-wise assessment of cluster stability. Computational Statistics and Data Analysis 52: 258–271.

[56] Jeong H, Mason SP, Barabsi AL, Oltvai ZN (2001) Lethality and centrality in protein networks. Nature 411: 41–42. 11333967 10.1038/35075138

[57] Hahn MW, Kern AD (2005) Comparative genomics of centrality and essentiality in three eukaryotic protein-interaction networks. Molecular Biology and Evolution 22: 803–806. 15616139 10.1093/molbev/msi072

[58] He X, Zhang J (2006) Why do hubs tend to be essential in protein networks? PLoS Genet 2: e88. 16751849 10.1371/journal.pgen.0020088 PMC1473040

[59] Gething MJ, Sambrook J (1992) Protein folding in the cell. Nature 355: 33–45. 1731198 10.1038/355033a0

[60] Komander D (2009) The emerging complexity of protein ubiquitination. Biochemical Society transactions 37: 937–953. 19754430 10.1042/BST0370937

[61] Ikeda F, Dikic I (2008) Atypical ubiquitin chains: new molecular signals. ‘Protein modifications: Beyond the usual suspects’ review series. EMBO Reports 9: 536–542. 18516089 10.1038/embor.2008.93 PMC2427391

[62] Pickart CM, Fushman D (2004) Polyubiquitin chains: polymeric protein signals. Current Opinion in Chemical Biology 8: 610–616. 15556404 10.1016/j.cbpa.2004.09.009

[63] Wool IG (1979) The structure and function of eukaryotic ribosomes. Annual Review of Biochemistry 48: 719–754. 10.1146/annurev.bi.48.070179.003443 382996

[64] Yoshihama M, Nakao A, Nguyen HD, Kenmochi N (2006) Analysis of ribosomal protein gene structures: Implications for intron evolution. PLoS Genet 2: e25. 16518464 10.1371/journal.pgen.0020025 PMC1386722

[65] Saveanu C, Fromont-Racine M, Harington A, Ricard F, Namane A, et al (2001) Identification of 12 new yeast mitochondrial ribosomal proteins including 6 that have no prokaryotic homologues. The Journal of biological chemistry 276: 15861–15867. 11278769 10.1074/jbc.M010864200

[66] Graack HR, Wittmann-Liebold B (1998) Mitochondrial ribosomal proteins (MRPs) of yeast. Biochemical Journal 329: 433–448. 9445368 10.1042/bj3290433 PMC1219062

[67] Wieser R, Wrana JL, Massagu J (1995) GS domain mutations that constitutively activate t beta r-i, the downstream signaling component in the TGF-beta receptor complex. The EMBO journal 14: 2199–2208. 7774578 10.1002/j.1460-2075.1995.tb07214.x PMC398326

[68] Chen YG, Liu F, Massague J (1997) Mechanism of TGFbeta receptor inhibition by FKBP12. The EMBO journal 16: 3866–3876. 9233797 10.1093/emboj/16.13.3866 PMC1170011

[69] Massagu J (2000) How cells read TGF- signals. Nature Reviews Molecular Cell Biology 1: 169–178. 11252892 10.1038/35043051

[70] Shi Y, Massagu J (2003) Mechanisms of TGF-beta signaling from cell membrane to the nucleus. Cell 113: 685–700. 12809600 10.1016/s0092-8674(03)00432-x

[71] Annes JP, Munger JS, Rifkin DB (2003) Making sense of latent TGF activation. Journal of Cell Science 116: 217–224. 12482908 10.1242/jcs.00229

[72] Dijke Pt, Hill CS (2004) New insights into TGF-Smad signalling. Trends in Biochemical Sciences 29: 265–273. 15130563 10.1016/j.tibs.2004.03.008

[73] Sayed M, Pelech S, Wong C, Marotta A, Salh B (2001) Protein kinase CK2 is involved in g2 arrest and apoptosis following spindle damage in epithelial cells. Oncogene 20: 6994–7005. 11704824 10.1038/sj.onc.1204894

[74] Enserink JM, Kolodner RD (2010) An overview of cdk1-controlled targets and processes. Cell Division 5: 11. 20465793 10.1186/1747-1028-5-11 PMC2876151

[75] Chen J, Fujii K, Zhang L, Roberts T, Fu H (2001) Raf-1 promotes cell survival by antagonizing apoptosis signal-regulating kinase 1 through a MEKERK independent mechanism. Proceedings of the National Academy of Sciences 98: 7783–7788. 10.1073/pnas.141224398 PMC35419 11427728

[76] O'Neill E, Rushworth L, Baccarini M, Kolch W (2004) Role of the kinase MST2 in suppression of apoptosis by the proto-oncogene product raf-1. Science 306: 2267–2270. 15618521 10.1126/science.1103233

[77] Pei D, Zhang Y, Zheng J (2012) Regulation of p53: a collaboration between mdm2 and mdmx. Oncotarget 3: 228–235. 22410433 10.18632/oncotarget.443 PMC3359881

[78] Chen B, Fan W, Liu J, Wu FX (2013) Identifying protein complexes and functional modulesfrom static PPI networks to dynamic PPI networks. Briefings in Bioinformatics. 10.1093/bib/bbt039 23780996

[79] Tsai CJ, Ma B, Nussinov R (2009) Proteinprotein interaction networks: how can a hub protein bind so many different partners? Trends in Biochemical Sciences 34: 594–600. 19837592 10.1016/j.tibs.2009.07.007 PMC7292551

[80] Lancichinetti A, Fortunato S, Radicchi F (2008) Benchmark graphs for testing community detection algorithms. Phys Rev E 78: 046110. 10.1103/PhysRevE.78.046110 18999496

[81] Cafieri S, Hansen P, Liberti L (2014) Improving heuristics for network modularity maximization using an exact algorithm. Discrete Applied Mathematics 163, Part 1: 65–72.

[82] Shannon P, Markiel A, Ozier O, Baliga NS, Wang JT, et al (2003) Cytoscape: A software environment for integrated models of biomolecular interaction networks. Genome Research 13: 2498–2504. 14597658 10.1101/gr.1239303 PMC403769

