# Supplementary material for: Community Structure Detection for Overlapping Modules through Mathematical Programming in Protein Interaction Networks
Source: PLoS One. 2014 Nov 20;9(11):e112821. doi: 10.1371/journal.pone.0112821 (PMC4239042; doi:10.1371/journal.pone.0112821)
Supplement: File S1 — Supporting tables. Table S1, The significance values for the comparison of isolated nodes with connector nodes (topological features). Table S2, The significance values for the comparison of isolated nodes with connector nodes (functional features). Table S3, Essentiality results summary, where p-values less than 0.01 indicate that connector nodes in the corresponding organism are significantly enriched for essential genes. Table S4, The Jaccard index for each pair-wise comparison of sets of inter-connector nodes. Table S5, Summary of the significance values for the comparison between inter and intra-connectors based on topological measures. Table S6, Average participation coefficient for inter and intra-connectors with corresponding significance values. (PDF) [file pone.0112821.s001.pdf]

Community Structure Detection for Overlapping Modules through Mathematical Programming in Protein Interaction Networks
Supporting Information

Laura Bennett[1], Aristotelis Kittas[2], Songsong Liu[1],
Lazaros G. Papageorgiou[1] and Sophia Tsoka[2]

[1]Department of Chemical Engineering
University College London, Torrington Place, London
WC1E 7JE, United Kingdom

[2] Department of Informatics
King's College London, Strand, London
WC2R 7JE, United Kingdom

	**iMod**	**Louvain**	**QCUT**
**Rat**			
**Degree**	2.50E-32	2.19E-33	4.30E-31
**Betweenness**	1.87E-40	3.51E-40	7.22E-37
**Eigenvector**	7.57E-07	8.22E-07	3.99E-07
**E. coli**			
**Degree**	1.37E-29	7.51E-28	6.03E-27
**Betweenness**	2.62E-43	6.69E-41	6.05E-40
**Eigenvector**	2.25E-07	4.43E-07	4.06E-07
**Yeast**			
**Degree**	3.00E-40	2.36E-43	4.55E-46
**Betweenness**	5.28E-72	6.83E-67	1.34E-64
**Eigenvector**	1.37E-49	7.42E-53	8.36E-54
**Human**			
**Degree**	1.61E-260	$< 2.2e-16$	2.67E-292
**Betweenness**	$< 2.2e-16$	$< 2.2e-16$	$< 2.2e-16$
**Eigenvector**	1.53E-124	5.12E-173	2.42E-170

Table S1: The significance values for the comparison of isolated nodes with connector nodes (topological features).

	iMod	Louvain	QCUT
**Rat**			
**ALL GO**	8.70E-05	2.90E-04	1.29E-04
**MF**	1.35E-04	2.43E-03	1.19E-03
**BP**	2.01E-03	1.50E-03	1.11E-03
**CC**	4.06E-03	8.81E-03	7.66E-03
**Domains**	1.80E-01	1.96E-01	7.67E-02
**E. coli**			
**ALL GO**	6.50E-05	4.32E-05	4.32E-05
**MF**	5.72E-04	1.88E-04	1.88E-04
**BP**	3.57E-01	4.64E-01	4.64E-01
**CC**	6.41E-01	8.15E-01	8.15E-01
**Domains**	3.63E-03	2.35E-02	2.35E-02
**Yeast**			
**ALL GO**	9.63E-01	5.43E-01	2.10E-01
**MF**	3.31E-01	3.78E-01	8.63E-01
**BP**	3.96E-01	2.70E-01	1.12E-01
**CC**	1.20E-01	5.73E-01	5.99E-01
**Domains**	8.86E-06	3.47E-05	2.92E-04
**Human**			
**ALL GO**	4.59E-26	3.28E-28	3.97E-30
**MF**	2.18E-16	5.41E-19	1.24E-19
**BP**	3.84E-12	1.83E-15	1.04E-14
**CC**	7.14E-30	2.96E-27	3.78E-29
**Domains**	2.54E-10	2.03E-09	2.48E-09

Table S2: The significance values for the comparison of isolated nodes with connector nodes (functional features).

	**iMod**	**Louvain**	**QCUT**
**E. coli**	2.65E-04	9.99E-03	9.83E-04
**Yeast**	7.64E-04	1.11E-03	1.30E-03
**Human**	3.16E-15	1.31E-15	4.25E-19

Table S3: Essentiality results summary, where p-values less than 0.01 indicate that connector nodes in the corresponding organism are significantly enriched for essential genes.

**Network**	**Soft partitions**	**Jaccard**
	iMod+OverMod vs. Louvain+OverMod	0.7222
**Rat**	iMod+OverMod vs. QCUT+OverMod	0.7647
	Louvain+OverMod vs. QCUT+OverMod	0.6607
	iMod+OverMod vs. Louvain+OverMod	0.6721
**E. coli**	iMod+OverMod vs. QCUT+OverMod	0.8035
	Louvain+OverMod vs. QCUT+OverMod	0.7719
	iMod+OverMod vs. Louvain+OverMod	0.8767
**Yeast**	iMod+OverMod vs. QCUT+OverMod	0.7979
	Louvain+OverMod vs. QCUT+OverMod	0.8606
	iMod+OverMod vs. Louvain+OverMod	0.7786
**Human**	iMod+OverMod vs. QCUT+OverMod	0.7185
	Louvain+OverMod vs. QCUT+OverMod	0.7343

Table S4: The Jaccard index for each pair-wise comparison of sets of inter-connector nodes.

	**iMod+OverMod**	**Louvain+OverMod**	**QCUT+OverMod**
**Rat**			
Degree	2.14E-04	2.54E-03	7.90E-04
Betweenness	1.22E-02	1.09E-01	1.31E-01
Eigenvector	1.07E-02	4.05E-02	1.91E-02
**E. coli**			
Degree	1.48E-03	4.07E-04	8.94E-04
Betweenness	8.22E-02	7.51E-03	3.99E-02
Eigenvector	1.53E-02	1.14E-02	2.90E-03
**Yeast**			
Degree	1.02E-01	5.48E-02	1.06E-02
Betweenness	2.85E-04	7.03E-03	1.95E-03
Eigenvector	1.96E-03	1.38E-02	1.55E-03
**Human**			
Degree	3.09E-19	2.41E-03	4.58E-14
Betweenness	2.24E-08	2.93E-01	2.13E-07
Eigenvector	6.14E-06	2.13E-02	4.77E-01

Table S5: Summary of the significance values for the comparison between inter and intra-connectors based on topological measures.

	**iMod+OverMod**			**Louvain+OverMod**			**QCUT+OverMod**		
	Inter	Intra	p-value	Inter	Intra	p-value	Inter	Intra	p-value
**Rat**	0.4409	0.2818	7.24E-05	0.4409	0.2721	1.74E-04	0.4599	0.2685	2.11E-05
**E. coli**	0.3851	0.2176	4.33E-03	0.3892	0.2181	1.69E-03	0.3946	0.2137	1.78E-03
**Yeast**	0.3073	0.1441	7.93E-10	0.3079	0.1562	2.32E-06	0.3006	0.1607	2.12E-06
**Human**	0.5465	0.3188	1.81E-115	0.5351	0.3541	2.06E-107	0.5234	0.3443	3.09E-135

Table S6: Average participation coefficient for inter and intra-connectors with corresponding significance values.
